# Mechanistic Insight into the Effects of Curcumin on Neuroinflammation-Driven Chronic Pain

**DOI:** 10.3390/ph14080777

**Published:** 2021-08-07

**Authors:** Hasriadi, Peththa Wadu Dasuni Wasana, Opa Vajragupta, Pornchai Rojsitthisak, Pasarapa Towiwat

**Affiliations:** 1 Pharmaceutical Sciences and Technology Program, Faculty of Pharmaceutical Sciences, Chulalongkorn University, Bangkok 10330, Thailand; adhiehasri@gmail.com (H.); dasuniwasana@ahs.ruh.ac.lk (P.W.D.W.); 2 Research Affairs, Faculty of Pharmaceutical Sciences, Chulalongkorn University, Bangkok 10330, Thailand; opa.vaj@mahidol.ac.th; 3 Natural Products for Ageing and Chronic Diseases Research Unit, Chulalongkorn University, Bangkok 10330, Thailand; pornchai.r@chula.ac.th; 4 Department of Food and Pharmaceutical Chemistry, Faculty of Pharmaceutical Sciences, Chulalongkorn University, Bangkok 10330, Thailand; 5 Department of Pharmacology and Physiology, Faculty of Pharmaceutical Sciences, Chulalongkorn University, Bangkok 10330, Thailand

**Keywords:** chronic pain, curcumin, neuroinflammation, microglia, astrocyte

## Abstract

Chronic pain is a persistent and unremitting condition that has immense effects on patients’ quality of life. Studies have shown that neuroinflammation is associated with the induction and progression of chronic pain. The activation of microglia and astrocytes is the major hallmark of spinal neuroinflammation leading to neuronal excitability in the projection neurons. Excessive activation of microglia and astrocytes is one of the major contributing factors to the exacerbation of pain. However, the current chronic pain treatments, mainly by targeting the neuronal cells, remain ineffective and unable to meet the patients’ needs. Curcumin, a natural plant product found in the Curcuma genus, improves chronic pain by diminishing the release of inflammatory mediators from the spinal glia. This review details the role of curcumin in microglia and astrocytes both in vitro and in vivo and how it improves pain. We also describe the mechanism of curcumin by highlighting the major glia-mediated cascades in pain. Moreover, the role of curcumin on inflammasome and epigenetic regulation is discussed. Furthermore, we discuss the strategies used to improve the efficacy of curcumin. This review illustrates that curcumin modulating microglia and astrocytes could assure the treatment of chronic pain by suppressing spinal neuroinflammation.

## 1. Introduction

Chronic pain is clinically characterized by pain hypersensitivity (allodynia and hyperalgesia) and spontaneous pain lasting for months or years [1]. Chronic pain afflicts millions of people worldwide and remains prevalent due to its complex pathogenesis [2]. Chronic pain is regulated by neuronal and non-neuronal mechanisms in the peripheral and central nervous systems. The neuronal mechanism is characterized by peripheral and central sensitization, whereas the non-neuronal mechanism is manifested by the activation of immune cells [3,4]. Specifically, the activation of non-neuronal cells in the central nervous system (CNS), including microglia and astrocytes, which is known as neuroinflammation, plays a critical role in the initiation and maintenance of chronic pain [5]. Existing pharmacotherapy for chronic pain includes acetaminophen, opioid analgesics, steroids, non-steroidal anti-inflammatory drugs (NSAIDs), anti-depressants, anticonvulsants, and topical agents containing conventional analgesics [6]. However, the modest efficacy, temporary relief, and side effects of most existing drugs limit the therapeutic outcome [7]. Therefore, continuous efforts are required to develop new analgesic drugs, especially those targeting microglia and astrocytes, which are critical players in chronic pain.

Chronic pain driven by neuroinflammation is clinically relevant. Neuroinflammation associated with pain has been found in patients with chronic low back pain, fibromyalgia, complex regional pain syndrome (CRPS), osteoarthritis, and chronic radicular pain [8,9,10,11]. Animal studies have demonstrated that chronic pain arises from the activation of the microglia and astrocytes in the dorsal horn of the spinal cord, leading to increased production of pro-inflammatory mediators [12]. The release of these inflammatory mediators by microglia and astrocytes initiate and enhances pain transmission via the activation of projection neurons [13]. Glial cell modulators may substantially reduce the release of pro-inflammatory cytokines and increase the release of anti-inflammatory cytokines, as well as improve the pain state and overall quality of life of the patients. Specifically, they modify the balance between anti- and pro-inflammatory cytokines. Multiple glial cell modulators, such as fluorocitrate, ibudilast, minocycline, naltrexone, and propentofylline [14,15], have been assessed in clinical trials. However, the development of drugs targeting neuroinflammation is still in its infancy and needs to be further advanced. In this regard, natural compounds such as curcumin may play a pivotal role in advancing drug development targeting neuroinflammation, supported by the robust literature that evidence its ability to suppress neuroinflammation both in vitro and in vivo.

Curcumin, a polyphenolic compound commonly found in *Curcuma* spp., can target microglia and astrocytes and prevent the release of pro-inflammatory mediators [16,17]. Curcumin has been shown to alleviate spinal neuroinflammation in many pain models. Curcumin significantly reduces pain-like behaviors, such as pain hypersensitivity and spontaneous pain, by suppressing neuroinflammation [18,19,20,21,22,23,24]. It also affects multiple pro-inflammatory mediator-dependent pathways associated with the improvement of the pain state. In addition, curcumin is recognized as a safe and well-tolerated compound [25,26]. Due to its pharmacological effectiveness and safety profile, curcumin has the potential to be tested in patients with chronic pain associated with neuroinflammation. Ample evidence suggests the promising effects of curcumin on multiple disorders, especially chronic pain, by alleviating the inflammation in the CNS. Accordingly, this review summarizes and highlights the involvement of curcumin in neuroinflammation-driven chronic pain. We also discuss how curcumin attenuates activation of microglia and astrocytes and release of inflammatory mediators in vitro and in vivo and its potential use in humans. Further, the strategies to improve the efficacy of curcumin, such as prodrug and drug formulation approaches, are discussed.

## 2. Overview of Curcumin

*Curcuma longa* L and other *Curcuma* spp. of the Zingiberaceae, or ginger family, are rich sources of curcumin. This plant is endemic in Southeast and South Asia and has been used as spices and herbs for centuries. Curcumin has been widely used in the food industry as a flavoring and coloring agent. It is a polyphenol [1,7-bis(4-hydroxy-3-methoxyphenyl)-1,6-heptadiene-3,5-dione] with pleiotropic properties [27]. It consists of two aromatic rings that contain a methoxy group connecting with a linker. Curcumin is a hydrophobic compound with low aqueous solubility [28]. Curcumin is also chemically unstable under alkaline conditions and when exposed to light [29,30,31].

Interestingly, curcumin has multiple pharmacological activities, including antioxidant, anticancer, antimicrobial, and anti-inflammatory [27,32]. However, its limitations, such as poor oral bioavailability leading to low curcumin content in the CNS, have been observed. Curcumin is easily degraded, rapidly metabolized, and poorly absorbed in the gastrointestinal tract [33,34]. Despite its low bioavailability and penetration level in the CNS, curcumin has been shown to have pharmacological activity in neuroinflammation-linked brain disorders in animal models. Curcumin was shown to alleviate the progression of chronic pain [17], Alzheimer’s disease [35], multiple sclerosis [36], Parkinson’s disease [37], and stroke [38].

## 3. Role of Glia in Neuroinflammation-Mediated Chronic Pain

Neuroinflammation is associated with the establishment and maintenance of chronic pain. Chronic pain is a highly complex disorder and a disease caused by several factors, including physical tissue injury, inflammation, metabolic disorder, neurotoxins, cancer, and chemotherapy-related neuropathies, which subsequently activate the resident immune cells in the spinal dorsal horn of the CNS [12]. In addition, the central immune cells in other parts of the brain, such as the anterior cingulate cortex, amygdala, medial prefrontal cortex, and hippocampus, are activated [39,40]. Thus, these cells, including microglia and astrocytes, are involved in the pathogenesis of neuroinflammation-mediated chronic pain.

Microglia are macrophage-like immune cells in the CNS that originated from the embryonic yolk sac. Microglia are in a ramified form in the inactivated state and become amoeboid when activated [41]. They are responsible for maintaining homeostasis, protecting the brain from pathogen phagocytes, and promoting neuronal survival, neurogenesis, synaptic refinement, and plasticity [42]. Microglia can be activated by several stimuli, such as toxins, damage-associated molecular patterns, pathogen-associated molecular patterns, and microorganisms. Additionally, microglia can be activated by the signals from other cells, such as neurons and astrocytes, via a well-known neuron-glia interaction [42,43]. In pathological conditions, resting microglia are transformed into M1 and M2 phenotypes. M1 phenotype of microglia increases the expression of pro-inflammatory mediators, while M2 phenotype plays a role in the expression of anti-inflammatory mediators. Despite microglial polarization of resting microglia to M1 or M2 phenotypes, M1 to M2 or M2 to M1 phenotypic switch also occurred in numerous types of neuroinflammatory disorders [44]. In chronic pain, microglial activation is characterized by a massive release of inflammatory mediators and the upregulation of numerous receptors, such as toll-like, purinergic, and chemokine receptors [12,45]. In addition, brain-derived neurotrophic factor (BDNF), one of the mediators released by microglia, plays a crucial role in neuron-microglia interaction. The excessive release of BDNF from microglia increased the pain transmission via modification of the anion reversal potential in the spinal neurons [46]. Other specific molecules expressed during microglial activation include the integrin cell adhesion molecule (Cd11b) and ionized calcium-binding adapter molecule 1 (Iba1) [12].

Akin to microglia, astrocytes are also involved in the regulation of spinal neuroinflammation. Astrocytes are abundant in the CNS [47]. In 1858, Rudolf Virchow, a German scientist, first discovered that astrocytes acted as connective tissues in the CNS [48]. Astrocytes originate from the radial glial cells in the neuroectodermal tube that migrate to the brain ventricles to form astrocytes. Astrocytes have multiple functions, such as modulation of neurotransmitters and the maintenance of BBB integrity, and the synaptic plasticity of neurons [49]. The activated astrocytes are grouped into A1 and A2 reactive astrocytes. A1 reactive astrocytes are characterized by the excessive release of cytokines, chemokines, proteases, lipid mediators, and growth factors. On the other hand, A2 reactive astrocytes likely have protective properties of the CNS [50,51]. Moreover, the transformation of A1 astrocytes to A2 and naive astrocytes is a continuum of astrocyte polarization states [52]. The activation of astrocytes is characterized by numerous markers, such as glial fibrillary acidic protein (GFAP), S100 protein, and glutamine synthetase [53].

**Figure 1:**
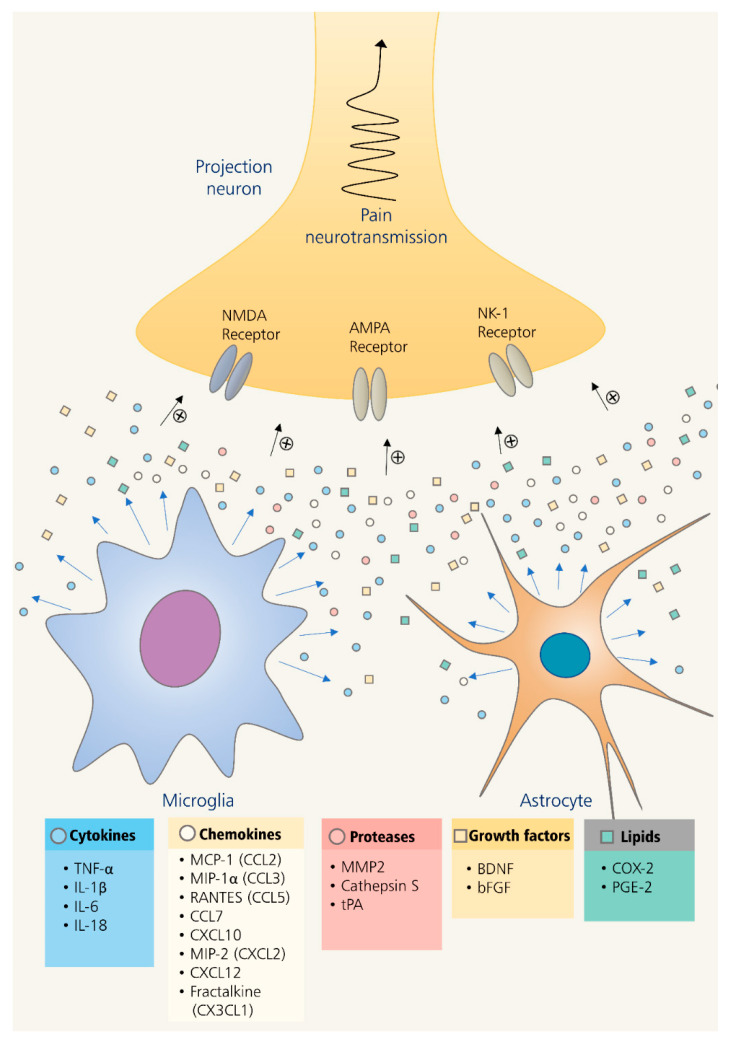
Pro-inflammatory mediators released by microglia and astrocytes activate projection neurons in the spinal cord. The mediators released by microglia and astrocytes include cytokines (TNF-α, IL-1β, IL-6, IL-18), chemokines (MCP-1, MIP-1α, RANTES, CCL7, CXCL10, MIP-2, CXCL12, fractalkine), proteases (MMP2, cathepsin S, tPA), growth factors (BDNF, bfGF), and lipids (COX-2, PGE2). Abbreviation: AMPA receptor, α-amino-3-hydroxy-5-methyl-4-isoxazole propionic acid receptor; BDNF, brain-derived neurotrophic factor; bfGF, basic fibroblast growth factor; CCL2, chemokine (C-C motif) ligand 2; CCL3, chemokine (C-C motif) ligand 3; CCL5, chemokine (C-C motif) ligand 5; CCL7, chemokine (C-C motif) ligand 7; COX-2; cyclooxygenase-2; CXCL2 chemokine (C-X-C motif) ligand 2; CXCL10, chemokine (C-X-C motif) ligand 10; CXCL12, chemokine (C-X-C motif) ligand 12; CX3CL1, chemokine (C-X3-C motif) ligand 1; IL-18, interleukin-18; IL-1β, interleukin-1β; IL-6, interleukin-6; MCP-1, monocyte chemoattractant protein-1; MIP-1α, macrophage inflammatory protein-1α; MIP-2, macrophage inflammatory protein-2; MMP2, matrix metalloproteinase-2; NK-1 receptor, neurokinin 1 receptor; NMDA receptor, N-methyl D-aspartate (NMDA) receptor; PGE2, prostaglandin E2; RANTES, regulated on activation, normal T cell expressed and secreted; TNF-α, Tumor necrosis factor-α; tPA, tissue-type plasminogen activator.

Chronic pain, mediated by the activation of microglia and astrocytes, is characterized by the massive release of neuroexcitatory substances and inflammatory mediators in the spinal cord [12,43]. Endogenous or exogenous signals activate these glia cells via inducing the pattern-recognition receptors. Numerous animal pain models, such as nerve injury, inflammatory pain, cancer pain, spinal cord injury, and paw incision, exhibit neuroinflammation in the spinal dorsal horn in parallel to increased pain-like behaviors [12]. Spinal neuroinflammation indicated by the activation of microglia and astrocytes is accompanied by the massive release of inflammatory mediators, including cytokines, chemokines, proteases, growth factors, and lipid (Figure 1) [12,13,54,55]. Within the spinal cord, pro-inflammatory mediators increase glutamatergic transmission and disinhibit the projection neurons [56]. Changes in the glutamatergic system are characterized by the release of excessive glutamate, upregulation of glutamate receptors, and downregulation of glutamate transporters in astrocytes, causing increased neuronal excitability in dorsal horn neurons. The spinal disinhibition is mediated by the downregulation of inhibitory neurotransmitters such as gamma-aminobutyric acid and glycine [57]. These mediators play a pivotal role in sensitizing the pain-processing neurons in the spinal cord in a process known as central sensitization, thus exacerbating pain perception in the brain cortex.

A growing body of evidence suggests the involvement of microglia and astrocytes in chronic pain. Modulating these non-neuronal cells in the CNS is a promising approach to abrogate chronic pain, as the existing therapies, which mainly target neuronal cells, have modest efficacy and are unable to meet the clinical need.

## 4. In Vitro Effects of Curcumin on Neuroinflammation

The effects of curcumin on microglial activation and the release of inflammatory mediators have been examined in primary cells and cell lines (Table 1). Lipopolysaccharides (LPS), lipoteichoic acid (LTA), trifluoroacetate salt (Pam3CSK4), gangliosides, cytokines, and interferon-gamma (IFNγ) are widely used to induce pro-inflammatory mediators and activate numerous signaling cascades.

In murine microglial BV-2 cells, curcumin exhibited anti-inflammatory activity against lipopolysaccharides (LPS)-induced neuroinflammation, as indicated by the reduced level of pro-inflammatory mediators, including nitric oxide (NO), inducible nitric oxide synthase (iNOS), cyclooxygenase (COX-2), prostaglandin E2 (PGE2), monocyte chemoattractant protein-1 (MCP-1), complement component 3, TNF-α (tumor necrosis factor-α), interleukin-1β (IL-1β) and interleukin-6 (IL-6) and the elevated level of endogenous anti-inflammatory mediators, including peroxisome proliferator-activated receptor alpha (PPAR-α), interleukin-4 (IL-4), and interleukin-10 (IL-10) [58,59,60,61,62]. In another study, HIVgp120, a glycoprotein of human immunodeficiency virus type I, was used to induce neuroinflammation in the microglial N9 cells; curcumin was shown to alleviate pro-inflammatory mediators, such as reactive oxygen species (ROS), TNF-α, and MCP-1 [63]. There is also ample evidence of curcumin’s inhibitory effect on the primary microglial cell on the release of LPS-induced inflammatory mediators, as verified by the reduced production of IL1-β, TNF-α, MCP-1, and macrophage inflammatory protein-1α (MIP-1α) [17]. In another study on a mixed culture of glial cells containing neurons and microglia, curcumin effectively reduced pro-inflammatory mediators, including CD11b (a marker of activated microglia), IL-1β, IL-6 and regulated on activation, normal T cell expressed and secreted (RANTES), against LPS-induced neuroinflammation, via inhibiting the activation of the nuclear factor kappa light-chain enhancer of activated B cells (NF-κβ) pathway [64]. Curcumin also supressed the expression of COX-2 and PGE2 in both BV-2 cells and primary microglia subjected to gangliosides, LPS, and IFNγ induced neuroinflammation by inactivating the Janus kinase-signal transducer and transcription activation by Janus kinase-signal transducer and activator of transcription (JAK-STAT) [65]. In line with this observation, curcumin was effective in attenuating NO, iNOS, TNF-α, COX-2, and PGE2 from either LTA- or Pam3CSK4-induced neuroinflammation by inactivating mitogen-activated protein kinase (MAPK) signaling and inducing the expression of the haem oxygenase-1 and nuclear factor erythroid 2-related factor 2 (Nrf2) [66,67]. Altogether, these findings strongly suggest glia-modulating anti-inflammatory effects of curcumin.

Moreover, numerous studies in cultured astrocytes indicate the potential of curcumin to attenuate inflammatory mediators (Table 2). Curcumin suppressed TNF-α, IL-1β, MCP-1, MIP-1α, and macrophage inflammatory protein-2 (MIP-2) in the primary astrocytes stimulated by LPS [17,68]. Moreover, curcumin repressed the expression of the COX-2 and IL-6 in IL1-β-stimulated primary cells by hindering the MAPK pathway [69]. In U-373 MG glioblastoma cells, curcumin reduced the LPS-stimulated expression of IL-6 and MCP-1 [70]. Curcumin also effectively decreased the induced production of GFAP, MCP-1, RANTES, CXCL10 by cytokines (TNF-α and IL-1β) in primary astroglial cells [71]. In addition, curcumin was shown to abolish NO production stimulated by LPS-IFNγ in C6 astroglial cells [72]. Moreover, an extended study in hydrogen peroxide-stimulated primary astroglial and human astrocytes-spinal cord cells revealed that curcumin consistently decreased intracellular ROS, GFAP, prdx6 and TNF-α production and substantially inhibited NF-κβ signaling and its nuclear translocation [73,74]. Regarding fructose-induced neuroinflammation, curcumin ameliorated activation of astrocytes by decreasing GFAP expression as wells as fractalkine and its receptors in a mixed culture of primary glial cells containing astrocytes, microglia and oligodendrocytes [75]. Furthermore, the NF-κβ pathway was activated in toxic metabolite (MMP^+^)-treated cells, thus stimulating astrogliosis and inflammation. Yet, curcumin’s presence attenuated the production of pro-inflammatory mediators TNF-α and IL-6 and increased the level of IL-10, an endogenous anti-inflammatory mediator [76]. These findings suggest that the pharmacological activity of curcumin is mediated via the effective regulation of both the pro-inflammatory and anti-inflammatory mediators.

## 5. In Vivo Effects of Curcumin on Neuroinflammation-Driven Chronic Pain

In rodents, curcumin has been found to alleviate pain-like behaviors in several chronic pain models, including neuropathic pain [21], radiculopathy [77], pain related to sickle cell disease [18], postoperative pain [78], osteoarthritis [79], trigeminal neuralgia [80], neuropathic pain in brachial plexus avulsion [19], diabetic neuropathy [23] and Complete Freund’s Adjuvant (CFA)-induced pain [17]. Specifically, some of them have shown the beneficial effects of curcumin in treating neuroinflammation-mediated chronic pain (Table 3). Curcumin affects the pain-like behaviors in rodents and can reduce the thermal and mechanical hypersensitivities as well as spontaneous pain. These results are consistent with reduced activation of microglia and astrocytes and the release of pro-inflammatory mediators in the spinal cord.

In well-characterized models of chronic neuropathic pain, such as chronic constriction injury (CCI) and spared nerve injury (SNI), curcumin attenuated pain-like behaviors, such as thermal and mechanical hypersensitivities, owing to the decreased activation of astrocytes and production of cytokines (TNF-α, IL-1β and IL-6), COX-2 and BDNF [21,22,81,82,83]. In chronic constriction injury (CCI) mice treated with oral curcumin 25–200 mg/kg/day for 14 days, curcumin attenuated the mechanical and thermal hyperalgesia along with IL-6 and TNF-α expression in the spinal cord at the doses 50 mg/kg and above, where the 25 mg/kg dose was ineffective [83]. However, in the oxaliplatin-induced neuropathic pain model, 12.5–50 mg/kg/day oral curcumin treatment for 28 days considerably improved the pain-like behaviors. This improvement was associated with reduced pro-inflammatory mediators such as TNF-α, IL-1β, and IL-6 and increased endogenous antioxidants [24]. These two findings indicate the efficacy of orally administered curcumin at two different dose levels where the discrepancies could be due to the differences in the pain model and the duration of curcumin administration.

In one study, CFA-induced inflammation was used to mimic arthritis in rats by injecting CFA to the plantar surface of the hind paws; intrathecal curcumin (0.1 mg and 1 mg) significantly improved the rats’ pain hypersensitivity, decreased activation of microglia and astrocytes (CD11b and GFAP) and hindered the release of pro-inflammatory mediators, IL-1β, TNF-α, MCP-1, and MIP-1α, from the astrocytes and microglia [17]. However, short-term, 2-day treatment of 100 mg/kg curcumin intraperitoneally did not significantly reduce inflammatory cytokines in the spinal cord of the CFA-induced rats [84]. This indicates the necessity of using alternative routes to deliver curcumin to obtain optimum effects.

**Figure 2:**
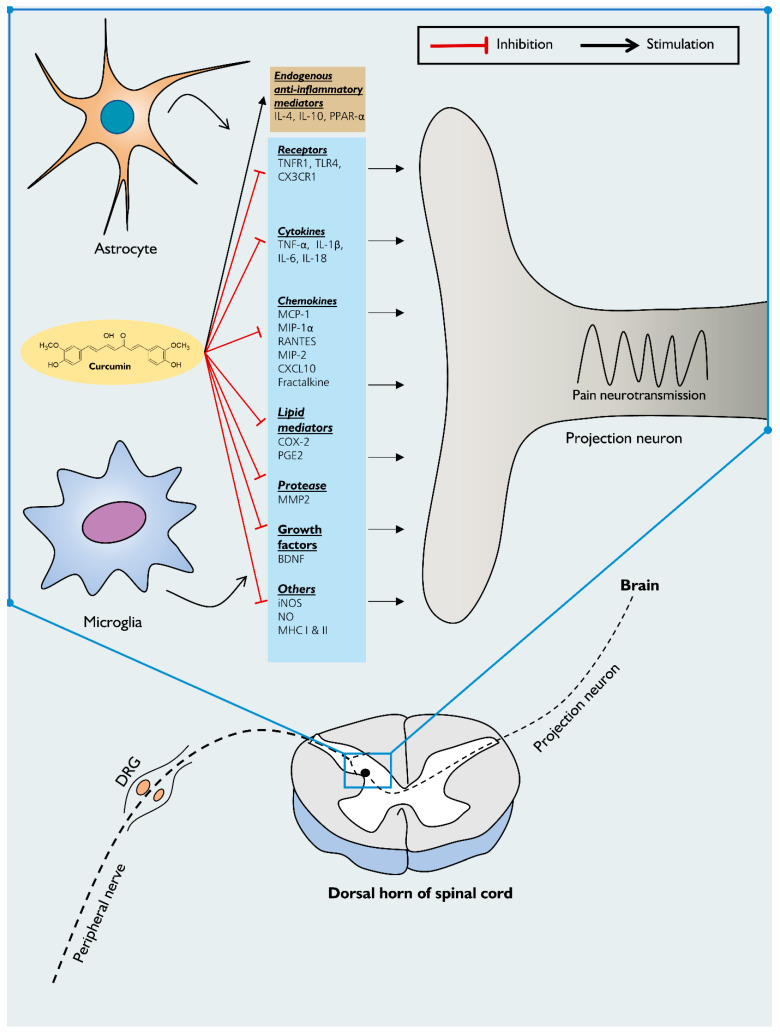
Curcumin suppresses pro-inflammatory mediators and enhances endogenous anti-inflammatory mediators released by spinal glia, resulting in decreased pain transmission by projection neurons. Curcumin inhibits the spinal glia-mediated neuroinflammation by inhibiting the release of pro-inflammatory mediators, including cytokines, chemokines, lipid mediators (COX-2 and PGE2), MMP2, BDNF, iNOs, and NO. It also increases the release of anti-inflammatory mediators, including IL-4, IL-10, and PPAR-α. At the receptor level, curcumin impedes TNFR1, TLR4, and CX3CR1 receptors. Through these mechanisms, curcumin limits the neuron-glia crosstalk, inhibiting central sensitization. Abbreviations: BDNF, brain-derived neurotrophic factor; COX-2, cyclooxygenase-2; CX3CR1, CX3C chemokine receptor 1; CXCL10, chemokine (C-X-C motif) ligand 10; DRG, dorsal root ganglia; IL, interleukin; iNOS, inducible nitric oxide synthase; MCP-1, monocyte chemoattractant protein-1; MHC, major histocompatibility complex; MIP-2, macrophage inflammatory protein 2; MIP-1α, macrophage inflammatory protein 1-alpha; NO, nitrite oxide; PPAR-α, peroxisome proliferator-activated receptor-α; TNFR1, tumor necrosis factor receptor 1; TLR4, toll-like receptor 4; TNF-α, tumor necrosis factor-alpha; PGE2, prostaglandin E2; MMP2, matrix metalloproteinase-2; RANTES, regulated on activation, normal T cell expressed and secreted.

In rats with spinal cord injury (SCI), curcumin (100 mg/kg, i.p) increased their locomotor activity compared to the control, indicating its effectiveness in improving spontaneous pain behaviors [20]. In addition, both acute and chronic administration of curcumin at a dose of 40 mg/kg (i.p) decreases spinal neuroinflammation, as characterized by reduced activation of astrocytes and the production of the inflammatory mediators, including BDNF, RANTES, and iNOS [21,85,86]. The data available in Table 3 indicate the ability of curcumin to alleviate neuroinflammation-mediated chronic pain in different rodent models (Figure 2). Yet, it is difficult to compare the effect of curcumin in rodent models of neuroinflammation-driven chronic pain due to the variability between models, route of administration, different dose regimens, and different animal species used.

Curcumin has not only direct effects on microglia and astrocytes in the central nervous system but also indirect effects through the regulation of peripheral nerves and immune cells. The direct effects of curcumin in the CNS are reasonable as many studies reported that curcumin reaches the CNS, including in the brain and spinal cord [87]. On the other hand, the potential effect of curcumin through indirect action remains unclear. Curcumin improved pain-like behaviors by regulating the transient receptor potential vanilloid 1 (TRPV1) of sensory neurons and purinergic and CX3C chemokine receptor 1 (CX3CR1) receptors of dorsal root ganglia [88,89,90,91]. Regulation of these receptors by curcumin may limit neuron-glia crosstalk leading to inhibition of spinal neuroinflammation. Previous studies reported that peripheral immune cells and central immune cells possess crosstalk in pain conditions [92]. Accordingly, the activation of peripheral immune cells can stimulate the activation of microglia and astrocytes [93,94]. As such, the activity of curcumin on neuroinflammation-driven chronic pain can also be through the modulation of peripheral immune cells, as many studies reported the ability of curcumin to modulate peripheral immune cells and dampens their inflammatory mediator releases [95]. The modulation of peripheral immune cells by curcumin may limit crosstalk between peripheral and central immune cells, which then limit neuroinflammation-driven chronic pain conditions.

In addition, the involvement of the gut microbiome in the development of neuroinflammation-associated pain has been reported and widely discussed yet not fully understood [96]. The crosstalk between the gut microbiome and central immune cells, including microglia and astrocytes, contributes to the changes in the CNS [96,97]. Regarding the potential activity of curcumin, a previous study reported that curcumin modulated gut microbiota in rats fed with a high-fat diet [98]. In an acute ileitis model, curcumin was also found to improve acute small intestinal inflammation in mice by increasing anti-inflammatory lactobacilli and bifidobacteria loads and decreasing pro-inflammatory enterobacteria and enterococci [99]. The ability of curcumin to modulate peripheral immune cells and gut microbiota might play a role in reducing neuroinflammation-associated pain. However, there is limited evidence to support these hypotheses. Therefore, future studies are warranted.

**Table 1:** Overview of the effect of curcumin on the activated microglia assayed in cultured cells.

No	Cell	Exposure	Concentration (µM)	Effects	Reference
1	Rat primary microglia and BV-2 microglial cells	50 µg/mL gangliosides, 100 ng/mL LPS, and 10 U/mL IFNγ	5–10	↓ COX-2 and iNOS	(H. Y. Kim et al., 2003) [65]
				↓ JAK-STAT signaling pathway	
2	BV-2 microglial cells	0.2 ng/mL LPS	2–16	↓ COX-2 production	Kang et al., 2004) [59]
				↓ NF-κβ signaling and AP-1 binding activity	
3	BV-2 microglial cells	0.5 μg/mL LPS	10–20	↓ NO, iNOS, COX-2, PGE2, TNF-α, IL1-β, and IL-6	(C.-Y. Jin et al., 2007) [58]
				↓ NF-κβ signaling pathway	
4	BV-2 microglial cells	100 ng/mL LPS	20	↑ the expression of IL-4 and PPAR-α	(Karlstetter et al., 2011) [62]
				↓ TLR2 and PGE2	
				↓ the expression of C3, NOS2, STAT1, MCP-1	
5	N9 microglial cells	1 µg/mL HIV-1 gp120	15	↓ ROS, TNF-α and MCP-1 productions	(Guo et al., 2013) [63]
6	Primary cells containing neurons and microglia	1 μg/mL LPS	2	↓ TLR4, MyD88, CD11b, IL-1β, IL-6, and RANTES	(H. Zhu et al., 2014) [64]
				↓ NF-κβ signaling pathway	
7	Primary microglial cells	1 μg/mL LPS	10–25	↓ IL1-β, TNF-α, MCP-1, MIP-1α	(J.-J. Chen et al., 2015) [17]
8	BV-2 microglial cells	1 μg/mL LPS	10–50	↓ NO, iNOS, TNF-α, IL-1β, and IL-6	(Cianciulli et al., 2016) [60]
				↓ PI3K/Akt and NF-κβ signaling pathways	
9	BV-2 microglial cells	0.1 μg/mL Pam3CSK4	10–20	↓ NO, TNF-α, NOS2, COX-2, PGE2	M. Jin et al., 2018) [66]
				↑ HO-1 and Nrf2 expression	
				↓ NF-κβ and MAPK signaling pathways	
10	BV-2 microglial cells	5 μg/mL LTA	10–20	↓ TNF-α, NO, NOS2,COX-2, and PGE2	(Y. Yu et al., 2018) [67]
				↑ HO-1 and Nrf2 expression	
				↓ MAPK signaling pathways	
11	BV-2 microglial cells	1 μg/mL LPS	10–50	↑ anti-inflammatory cytokines (IL-4 and IL-10) and ↑ SOCS-1	(Porro et al., 2019) [61]
				↓ JAK-STAT signaling pathway	

Abbreviations: ↓, decrease; ↑ increase; AP-1, activator protein 1; C3, complement C3; CD11b, cluster of differentiation molecule 11B; COX-2, cyclooxygenase-2; HIV-1 gp120, HIV-1 envelope protein gp120; HO-1, heme oxygenase-1; IL-10, interleukin 10; IL-1β, interleukin 1 beta; IL-4, interleukin 4; IL-6, interleukin 6; iNOS, inducible nitric oxide synthase; IFNγ, interferon gamma; JAK-STAT, the Janus kinase-signal transducer and activator of transcription; LPS, lipopolysaccharides; LTA, lipoteichoic acid; MAPK, the mitogen-activated protein kinase; MCP-1, monocyte chemoattractant protein-1; MIP-1α, macrophage inflammatory protein 1-alpha; MyD88, myeloid differentiation primary response 88; NF-κβ, nuclear factor κ light-chain-enhancer of activated B cells; NO, nitric oxide; NOS2, nitric oxide synthase 2; Nrf2, the nuclear factor erythroid 2-related factor 2; Pam3CSK4, Pam3Cys-Ser-(Lys)4 (trifluoroacetate salt); PGE2, prostaglandin E2; PI3K/Akt, phosphatidylinositol 3-kinase/protein kinase B; PPAR-α, peroxisome proliferator-activated receptor; RANTES, regulated on activation, normal T cell expressed and secreted; ROS, reactive oxygen species; SOCS-1, suppressor of cytokine signaling 1; STAT1, signal transducer and activator of transcription 1; TLR2, toll-like receptor 2; TLR4, toll-like receptor 4; TNF-α, tumor necrosis factor-alpha.

**Table 2:** Overview of the effect of curcumin on the activated astrocytes assayed in cultured cells.

No	Cell	Exposure	Concentration (µM)	Effects	Reference
1	C6 astroglial cells	6 pg/mL LPS 100 U/mL of IFNγ	-	↓ NO production	(Soliman and Mazzio 1998) [72]
2	Primary astrocytes	5 μg/mL LPS	10	↓ MIP-2 expression	(M. Tomita et al., 2005) [68]
3	Primary astrocytes	200 µM H_2_O_2_	10	↓ Intracellular ROS and ↑ activation of Nrf2 signaling pathways	(Jiang et al., 2011) [74]
4	C6 astroglial cells	1 μg/mL LPS	10–25	↓ MCP-1 and JNK pathway	(Z.-J. Zhang et al., 2012) [100]
5	U-373 MG cells	0.5 μg/mL LPS	5	↓ the expression of IL-6, MMP9 enzyme activity, MCP-1	(Seyedzadeh et al., 2014) [70]
6	Primary astrocytes	1 μg/mL LPS	10–25	↓ IL1-β, TNF-α, MCP-1, MIP-1α	(J.-J. Chen et al., 2015) [17]
7	Primary glial cells containing microglia, oligodendrocytes, and astrocytes	5 mM fructose	0.5–2	↓ the expression of fractalkine and its receptor, CX3CR1	(M. X. Xu et al., 2016) [75]
				↓ the GFAP, the marker of astrocyte activation	
8	Primary astrocytes	800 μM MPP^+^	8	↓ the release of TNF-α and IL-6	(S. Yu et al., 2016) [76]
				↑ the expression of IL-10	
				↓ the expression of TLR4 and inhibited NF-κβ pathway	
9	Primary astrocytes	20 ng/mL TNF-α + 10 ng/mL IL-1β	1	↓ GFAP, MCP-1, RANTES, CXCL10	(Yuan et al., 2017) [71]
				↓ NF-κβ signaling pathway	
10	Primary astrocytes	10 ng/mL IL-1β	10	↓ the expression of COX-2 and IL-6	(Drion et al., 2018) [69]
				↓ the activation of the MAPK pathways through p44 and p42	
				↓ the phosphorylation of ERK1/2	
11	Human Astrocytes-spinal cord	50 µM H_2_O_2_	1	↓ Intracellular ROS, GFAP, prdx6, TNF-α	(Daverey and Agrawal 2018) [73]
				↓ NF-κβ pathway and ↑ Nrf2 expression	

Abbreviations: ↓, decrease; ↑ increase; COX-2, cyclooxygenase-2; CX3CR1, CX3C chemokine receptor 1; CXCL10, chemokine (C-X-C motif) ligand 10; ERK1/2, extracellular signal-regulated protein kinases 1 and 2; GFAP, glial fibrillary acidic protein; IL-10, interleukin 10; IL-1β, interleukin 1 beta; IL-6, interleukin 6; IFNγ, interferon gamma; JNK, c-Jun N-terminal kinase; LPS, lipopolysaccharides; MAPK, the mitogen-activated protein kinase; MCP-1, monocyte chemoattractant protein-1; MIP-1α, macrophage inflammatory protein 1-alpha; MIP-2, macrophage inflammatory protein-2; MPP+, 1-methyl-4-phenylpyridinium; NF-κβ, nuclear factor κ light-chain-enhancer of activated B cells; NO, nitric oxide; Nrf2, the nuclear factor erythroid 2-related factor 2; prdx6, peroxiredoxin 6; RANTES, regulated on activation, normal T cell expressed and secreted; ROS, reactive oxygen species; TLR4, toll-like receptor 4; TNF-α, tumor necrosis factor-alpha; H_2_O_2_, hydrogen peroxide.

**Table 3:** Overview of the effect of curcumin on neuroinflammation-mediated chronic pain in animal models.

No	Animal	Animal Model of Pain	Dose (mg/kg/d)	Duration (Days)	Effects on Inflammatory Mediators in The Spinal Cord	Pain-Like Behaviors			References
						Pain Hypersensitivity		Spontaneous Pain	
						Thermal	Mechanical		
1	Sprague–Dawley rats	SIDN	60 mg/kg/d (p.o)	25	↓ TNF-α and TNFR1	✔	✔	N/A	(Y. Li et al., 2013) [23]
2	HbSS-BERK sickle mice	Sickle cell disease	15 mg/kg/d (p.o)	28	↓ the expression of Iba1 and GFAP, Substance P, and ROS	✔	✔	N/A	(Valverde et al., 2016) [18]
3	Sprague–Dawley rats	OIPN	12.5–50 mg/kg/d (p.o)	28	↑ SOD, GSH-Px, CAT, and ↓ MDA	✔	✔	N/A	(X. Zhang et al., 2020) [24]
					↓ TNF-α, IL-1β, IL-6, and NF-κβ activity				
4	ICR mice	CCI	25–200 mg/kg/d (p.o)	14	TNF-α and IL-6	✔	✔	N/A	(Limcharoen et al., 2021) [83]
5	Sprague–Dawley rats	SCI	40 mg/kg/d (i.p)	7	↓ GFAP expression	N/A	N/A	N/A	(M. S. Lin et al., 2011) [85]
6	Sprague–Dawley rats	SCI	40 mg/kg (i.p)	1	↓ RANTES and iNOS	N/A	N/A	N/A	(M.-S. Lin et al., 2011) [86]
7	Sprague–Dawley rats	CCI	100 mg/kg/(i.p)	14	↓ CX3CR1	✔	✔	N/A	Zheng et al., 2011) [91]
8	Sprague–Dawley rats	CCI	50 mg/kg/d (i.p)	7 and 14	↓ GFAP and phospho-ERK	✔	✔	N/A	(F. T. Ji et al., 2013) [82]
9	Sprague–Dawley rats	CCI	40 and 60 mg/kg/d (i.p)	7	↓ BDNF and COX-2, and P300/CBP HAT activity	✔	✔	N/A	(X. Zhu et al., 2014) [21]
10	Sprague–Dawley rats	CCI	100 mg/kg/d (i.p)	14	↓ NF-κβ p65 and CX3CR1	✔	✔	N/A	(Cao et al., 2014) [81]
11	Sprague–Dawley rats	SCI	100 mg/kg (i.p)	1	↓ TLR4, TNF-α, IL-1β, IL-6 and NF-κβ activity	N/A	N/A	✔	(Ni et al., 2015) [20]
12	Charles-Foster strain rats	CFA	100 mg/kg/d (i.p)	2	No effect on the cytokines	✔	N/A	N/A	(Singh and Vinayak 2015) [84]
13	BALB/c mice	SNI	60–120 mg/kg/0.5 d (i.p)	7	↓ GFAP, IL-1β, NALP1 inflammasome and JAK2-STAT3 signaling	✔	✔	N/A	(S. Liu et al., 2016) [22]
14	Sprague–Dawley rats	Brachial Plexus Avulsion	60 mg/kg/d (i.p)	28	↓ GFAP, TNF-α, IL-6, c-FOS, and NGF	✔	✔	N/A	(Xie et al., 2019) [19]
15	Sprague–Dawley rats	CFA	0.1 mg and 1 mg (i.t)	3	↓ IL-1β, TNF-α, MCP-1 and MIP-1α	✔	✔	N/A	(J.-J. Chen et al., 2015) [17]
16	ICR mice	CCI	CurDDG, 25–200 mg/kg/d (p.o)	14	↓ TNF-α and IL-6	✔	✔	N/A	(Limcharoen et al., 2021) [83]
17	ICR mice	CCI	CurDG, 25–200 mg/kg/d (p.o)	14	↓ TNF-α and IL-6	✔	✔	N/A	(Limcharoen et al., 2020) [101]
18	CD-1 mice	CCI	PLGA-CUR, 5–25 µg/mouse. (i.t)	1	↓ TNF-α, IL-β, IL-6 and BDNF	✔	✔	N/A	(Pieretti et al., 2017) [102]
19	ICR mice	OIH	PLGA-CUR 20 mg/kg, (p.o)	1	↓ spinal CaMKIIα	✔	✔	N/A	(Hu et al., 2016) [103]

Abbreviations: ↓, decrease; ↑ increase; ✔, improve; BALB/c, Bagg albino; BDNF, brain-derived neurotrophic factor; CaMKIIα, Ca^2+^/calmodulin-dependent protein kinase IIα; CAT, catalase; CCI, chronic constriction injury; CFA, complete Freund’s adjuvant; c-FOS, cellular oncogenes fos; COX-2, cyclooxygenase-2; CX3CR1, chemokine (C-X3-C motif) receptor 1; ERK, the extracellular-signal-regulated kinase; GFAP, glial fibrillary acidic protein; GSH-Px, glutathione peroxidase; Iba1, ionized calcium-binding adaptor molecule 1; ICR, institute of cancer research; IL-1β, interleukin 1 beta; IL-6, interleukin 6; iNOS, iNOS, inducible nitric oxide synthase; i.p, intraperitoneal administration; i.t, intratechal administration; JAK2-STAT3, Janus kinase 2-signal transducer and activator of transcription 3; MCP-1, monocyte chemoattractant protein-1; MDA, malondialdehyde; MIP-1α, macrophage inflammatory protein 1-alpha; N/A, not available; NALP1, NACHT leucine-rich-repeat protein 1; NF-κβ, nuclear factor κ light-chain-enhancer of activated B cells; NGF, nerve growth factor; OIPN, oxaliplatin-induced peripheral neuropathy; P300/CBP HAT, P300/CBP histone acetyltransferase; p.o, oral administration; RANTES, regulated on activation, normal T cell expressed and secreted; ROS, reactive oxygen species; SCI, spinal cord injury; SIDN, streptozotocin-induced diabetic neuropathy; SNI, spared nerve injury; SOD, superoxide dismutase; TLR4, toll-like receptor 4; TNFR1, tumor necrosis factor receptor 1; TNF-α, tumor necrosis factor-alpa; CurDDG, curcumin diethyl diglutarate; CurDG, curcumin diglutaric acid; PLGA-CUR, curcumin-loaded poly (lactide-co-glycolide) nanoparticles; OIH, opioid-induced hyperalgesia.

## 6. The Activation of Glial Cells in Humans with Chronic Pain and the Potential Use of Curcumin

Glial activation in patients with chronic pain has been demonstrated over the past years. For instance, integrated positron emission tomography magnetic resonance imaging revealed glial activation in multiple brain regions in patients with chronic low back pain [8]. In addition, positron emission tomography imaging of patients with fibromyalgia demonstrated increased brain glial activation [9]. Similarly, CRPS, characterized by severe and chronic pain, has activated glial cells in the thalamus [10]. Despite glial activation in the brain, spinal glial cells are modulated during chronic pain. A study of CRPS patients showed excessive spinal glial activation and neuronal loss [11].

Meanwhile, many studies have assessed the neuroinflammation in patients with chronic pain by analyzing the inflammatory mediators in the cerebrospinal fluid (CSF). Patients with peripheral neuropathic pain had higher levels of inflammatory markers and chemokines in CSF compared with the healthy group [104]. Similarly, the level of inflammatory mediators in CSF was increased in patients with osteoarthritis [105]. Moreover, patients with chronic radicular pain had increased inflammatory cytokines associated with neuro-immune interaction in the CSF [106]. The analgesic efficacy of curcumin has been tested in clinical trials. It was found that curcumin improves pain state in patients with osteoarthritis [107,108,109,110], rheumatoid arthritis [111,112], postoperative inflammation [113,114], and idiopathic inflammatory orbital pseudo-tumors [115]. While curcumin has been shown to improve pain clinically, its effect on neuroinflammation-associated chronic pain has not been examined. Though there has been considerable evidence of the role of curcumin on neuroinflammation in animal pain models in the last few years, its role in humans remains to be determined.

## 7. Mechanism of Action of Curcumin on Neuroinflammation-Driven Chronic Pain

### 7.1. MAPK Pathway

In general, the MAPK signaling pathway is involved in numerous cellular processes associated with cell proliferation and differentiation, cell migration, cell death, and immune response [116]. Studies have implicated the activation of the MAPK pathway in numerous chronic pain models, such as neuropathic, inflammatory, bone cancer, and diabetic neuropathic pain [12,43]. The kinases in the MAPK pathway are mainly classified into three categories, namely, extracellular signal-regulated kinases (ERKs), c-Jun N-terminal kinases (JNKs), and p38 [116]. Upon activation, extracellular ligands, including cytokines, chemokines, and gliotransmitters, transduce signals through the activated neurotransmitter receptors, growth factor receptors, chemokines, and cytokines receptors and activate the ERK, JNK, and p38 via phosphorylation [117]. Chronic pain can be alleviated by modulating glial cells via the MAPK pathway. MAPK inhibitors have been shown to have an analgesic effect on chronic pain both in pre-clinical and clinical studies [15,118,119].

**Figure 3:**
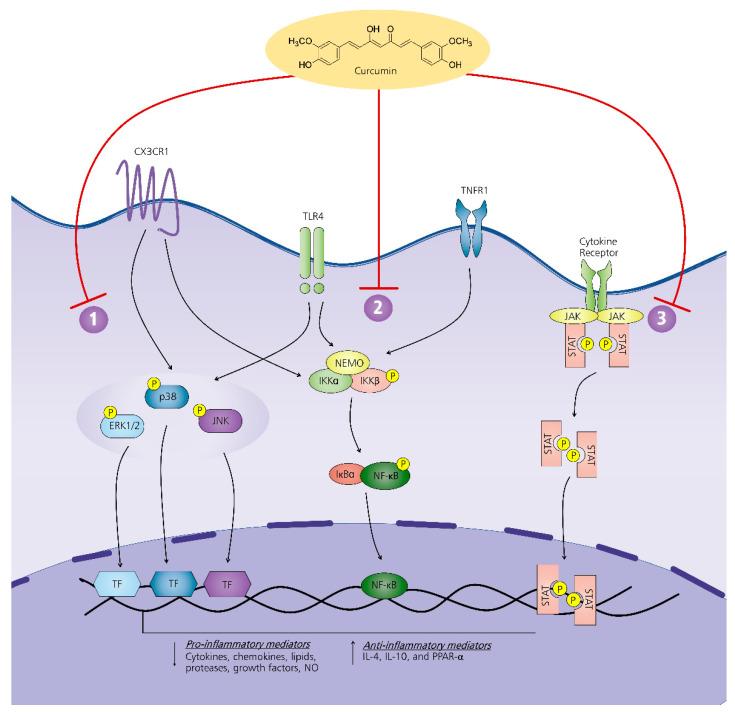
Proposed signaling cascades of curcumin in the spinal glia. Curcumin suppresses the activation of glial cells and inflammatory mediators via modulating the (1) MAPK, (2) NF-κβ, and (3) JAK-STAT pathways. (1) Curcumin regulates the MAPK signaling pathway by suppressing the activation of three major kinases: ERK, p38, and JNK, and at the upstream level, curcumin reduces the expression of CX3CR1 and TLR4 receptor that inhibits the activation of p38 in the MAPK pathway. (2) Curcumin inhibits the NF-κβ pathway via abrogating activation of NF-κβ. At the upstream level NF-κβ pathway, curcumin inhibits the expression of CX3CR1, TLR4, and TNF-α receptors. (3) Curcumin inhibits neuroinflammation by suppressing the phosphorylation of JAK and STAT proteins. Suppression of these pathways inhibits the release of pro-inflammatory mediators and enhances the release of anti-inflammatory mediators by spinal glia. Abbreviations: CX3CR1, CX3C chemokine receptor 1; TLR4, toll-like receptor 4; TNFR1, tumor necrosis factor receptor 1; ERK, extracellular-signal-regulated kinase, JNK, c-Jun N-terminal kinases; TF, transcription factor; NEMO, NF-κβ essential modulator, IKK, IκB kinase; NF-κβ, nuclear factor κ light-chain-enhancer of activated B cells; JAK, Janus kinase; STAT, signal transducer and activator of transcription protein; NO, nitrite oxide; IL, interleukin, PPAR-α, peroxisome proliferator-activated receptor-α.

Multiple studies have observed that curcumin modulates microglia and astrocytes by inhibiting the MAPK pathway. In the upstream pathway, curcumin decreased the expression of CX3CR1 and toll-like receptor 4 (TLR4) receptors that are involved in the activation of the p38 MAPK pathway [75,120]. In a rat model of neuropathic pain, fractalkine mediated the activation of spinal microglia via its receptor CX3CR1, leading to activation of its downstream pathway, p38 MAPK [121]. Furthermore, the activation of the TLR4 receptor in the spinal cord has also been associated with the activation of p38 MAPK, leading to overexpression of IL-6 and BDNF in a rat model of cancer-induced bone pain [122]. In cultured cells, curcumin diminished the activation of MAPK pathways in both astrocytes and microglial cells. In LPS-induced astroglial cells, curcumin reduced the expression of inflammatory markers via the suppression of the p-JNK and ERK1/2 pathways [69,100]. Moreover, curcumin repressed MAPK phosphorylation in the BV-2 cells induced by Pam3CSK4 and LTA [66,67]. Consistent with the data from cultured cells, the repeated administration of curcumin attenuated pain hypersensitivity and astrocyte activation by lowering ERK phosphorylation in a rat model of neuropathic pain [82]. Hence, the MAPK pathway is considered a potential target of curcumin to inhibit neuroinflammation-mediated pain (Figure 3).

### 7.2. NF-κβ Pathway

The NF-κβ signaling pathway is primarily involved in regulating inflammatory responses and the production of pro-inflammatory mediators [123]. NF-κβ is a pivotal regulator of spinal neuroinflammation and is significantly involved in the induction and progression of chronic pain. In rodent models of neuropathic pain, the NF-κβ pathway was activated, thus increasing pain hypersensitivity [124,125].

The NF-κβ pathway is classified into canonical and non-canonical pathways. An inflammatory response usually occurs via the canonical pathway, which is activated by several ligands, such as cytokines, chemokines, pathogen-associated molecular patterns, and danger-associated molecular patterns [126]. These ligands activate the receptors, including toll-like receptors such as TLR4 and TLR2, purinergic receptors, cytokine receptors, and chemokine receptors. Once a ligand binds to the receptor, the IKK kinase (IKK) complex containing IKKα, IKKβ, and NEMO is phosphorylated by protein kinase followed by the degradation of Iκβs (Iκβα, Iκββ or Iκβε), leading to the translocation of NF-κβ to the nucleus [123,126].

While transcriptional NF-κβ monomers, consisting of p50 (NF-κβ 1), p52 (NF-κβ2), p65 (RelA), c-Rel, and Rel-B, form homodimers and heterodimers [123], the p65-p50 NF-κβ heterodimer is mostly found during inflammation [127]. In the basal condition, NF-κβ dimers are in the cytoplasm and interact with Iκβs, their negative regulators. However, NF-κβ is activated by phosphorylation by protein kinase A (PKAc) at its Ser/Thr/Tyr residue, specifically in the Rel homology domain and C-terminal transactivation domain. After the activated NF-κβ translocates to the nucleus, p65 is phosphorylated by mitogen- and stress-activated kinases 1 and 2 to regulate the expression of inflammatory mediators. In addition, the post-translational modification of NF-κβ, including phosphorylation, acetylation, and methylation, contributes to the activation of the NF-κβ pathway [128].

In chronic pain, several approaches can be applied to inhibit the NF-κβ pathway, such as dephosphorylating NF-κβ, increasing the expression of Iκβ, maintaining the Iκβ-NF-κβ complex, and diminishing NF-κβ demethylation and deacetylation [127,129]. For instance, minocycline, a drug being tested in clinical trials, inhibits the NF-κβ pathway by impeding the expression of NF-κβ [130]. In addition, minocycline yielded promising results in decreasing pain behaviors in chronic pain patients [131], indicating that targeting glial cells via the NF-κβ pathway may result in significant pain relief. The NF-κβ pathway may also be blocked by modulating the receptors that activate this pathway, such as TLR2/4 and purinergic receptors and cytokine receptors [15].

In vitro and in vivo studies have shown that curcumin attenuates the activation of the NF-κβ signaling pathway. Curcumin was found to inhibit the upstream level of this pathway via downregulating the TLR4 receptor. Curcumin significantly reduced the protein levels of TLR4, Myd88, NF-κB p65, p-IκB-α, and Iba1 in mice with subarachnoid hemorrhage-related neuroinflammation together with the pro-inflammatory mediators, including TNF-α, IL-1β, IL-6, and iNOS [120]. In LPS-stimulated BV-2 cells, curcumin attenuated TLR4 expression and increased TREM2 expression, whereas, in the downstream level, curcumin decreased the expression of p-IκB-α and p-NF-κB p65 [132]. In the upstream level, curcumin was found to inhibit the expression of TNF-α receptor 1, a receptor involved in the activation of the NF-κβ pathway, along with its ligand (TNF-α) in streptozotocin-induced diabetic rats [23]. The activation of the TNF- α receptor induces the activation of transcription genes encoding pro-inflammatory mediators associated with pain [133]. Furthermore, the fractalkine/CX3CR1 axis-stimulated NF-κB pathway also plays a potential role in inflammatory pain [134,135]. With the curcumin treatment, this axis was attenuated, indicated by the downregulation of fractalkine and its receptor, CX3CR1, in fructose-fed mice with neuroinflammation [75]. In a ratmodel of neuropathic pain, curcumin improved mechanical and thermal hyperalgesia via inhibition of CX3CR1 expression in dorsal root ganglia and spinal cord [91]. Moreover, curcumin treatment reduced the level of the TLR4, p-IKK- α/β, p-Iκβ-α, and nucleic p65 and increased the expression of the Iκβ-α in the astrocytes in the spinal cord of ratswith spinal cord injury [20,71]. In rodent models of neuropathic pain, curcumin hindered the upregulation and phosphorylation of NF-κβ [24,81]. In a mixed culture of glial cells induced by LPS, curcumin attenuated the level of TLR4, MyD8, p-Iκβ-α, and p65 and upregulated the Iκβ-α, indicating the suppression of the TLR4/MyD88/NF-κβ cascade by curcumin [64]. In the activated BV-2 microglial cells, curcumin also inhibited the NF-κβ cascade by impeding the phosphorylation of Iκβ-α and p65 [58,59,66].In addition, one study showed curcumin repressed the expression of the TLR4 and NF-κβ [76]. Both in vitro and in vivo studies demonstrated that curcumin reduces pro-inflammatory mediators and decreases pain-like behaviors via the NF-κβ pathway (Figure 3).

### 7.3. JAK-STAT Pathway

The JAK-STAT pathway is a critical regulator of cell fate and immune response [136]. In several pain models, the activation of the JAK-STAT pathway plays a pivotal role in the maintenance and progression of chronic pain by regulating the immune response in the spinal cord. In the spinal cord of CCI rats, the JAK-STAT3 pathway was activated through the phosphorylation of STAT3, leading to neuroinflammation as characterized by the increased expression of the suppressor of cytokine signaling 3 (Socs3), IL-6, MCP-1, and the integrin alpha M (Itgam), a marker of microglial activation [137]. In spinal nerve ligated rats, the JAK-STAT3 pathway was activated by IL-6 in the dorsal spinal horn, subsequently accumulating phosphor-STAT and increasing the expression of STAT3 target genes such as *Socs3* and *Cox-2*. This study also confirmed that the JAK2 inhibitor administered to rats could significantly reduce pain behaviors [138]. In the spinal nerve injury model, the activation of JAK-STAT was characterized by cell proliferation and astrogliosis [139]. In addition, oxaliplatin-induced neuropathic pain in rats showed overexpression of p-STAT and IL-6 and increased pain hypersensitivity; these patterns were then reversed by a JAK inhibitor, AG490 [140]. Thus, targeting the JAK-STAT3 pathway will help to lower the inflammatory mediators produced by microglia and astrocytes and may have a significant impact on chronic pain treatment.

JAK family consists of Janus Kinase JAK 1 (JAK1), Janus Kinase 2 (JAK2), Janus Kinase 3 (JAK3), and tyrosine kinase 2 (TYK2). Ligands of this pathway, including cytokines, interleukins, and growth factors, bind to transmembrane receptor families (type I and II receptors), leading to trans- or autophosphorylation of the inactive kinases [136,141]. The Rac-GTPase-activating protein then activates these dimers to translocate to the nucleus and bind to DNA regulatory elements [142]. The type I receptor (γ-chain) is usually triggered by IL-2, IL-4, IL-7, IL-9, IL-15, and IL-21 through JAK1 and JAK3 phosphorylation continued with the activation of STAT 5A, 5B, and 6. In contrast, the type II receptor is activated by IL-19, IL-20, IL-22, and IL-26, which then continued by the activation of JAK1, JAK2, TYK2, and STAT3 [143,144].

Curcumin lowered the activation of the JAK-STAT pathway in glial cells, not only in cultured cells but also in animal models. In a mouse model of neuropathic pain, curcumin treatment alleviated pain hypersensitivity, diminished astrogliosis, and reduced IL-1β expression via the JAK-STAT3 signaling pathway. The inhibition in phosphorylation of JAK2 and STAT3 resulted in the decreased IL-1β production [22]. Curcumin is also effective in alleviating astrogliosis and the activation of the JAK-STAT cascade in rats with spinal cord injury [145]. In the activated microglia, curcumin suppressed the production of pro-inflammatory mediators, COX-2 and iNOS. It also enhanced the expression of endogenous anti-inflammatory cytokine IL-4 and IL-10 via lowering the expression of JAK1/2 and STAT1/3 and the phosphorylation of JAK1/2 as well as STAT1/3 [61,65]. These findings strongly suggest that curcumin decreases pro-inflammatory mediators, increases endogenous anti-inflammatory cytokines, and improves pain hypersensitivity via suppressing the JAK-STAT pathway (Figure 3).

## 8. Role of Curcumin on Epigenetic Modulation and Inflammasome on Neuroinflammation-Driven Chronic Pain

There is growing evidence that neuroinflammation-associated epigenetic modification is involved in the induction and development of chronic pain [146]. Epigenetic modifications are inheritable modifications in gene expression that do not require changes in the DNA sequence. Pain caused by tissue injury, toxins, and environmental factors causes epigenetic changes, such as DNA methylation, histone acetylation, and RNA interference, subsequently altering the gene expression linked with pro-inflammatory mediators [146,147]. Epigenetic modifications have also been reported to control the transition from acute pain to chronic pain [147].

Epigenetic modifications also play an essential role in spinal neuroinflammation. There is growing evidence on the role of curcumin as an epigenetic modulator. At cellular levels, curcumin modulates epigenetic machinery by regulating DNA methyltransferase, histone modification, and mRNA [148]. Specifically, several studies on the effect of curcumin on epigenetic modulation have been performed in immune cells. Curcumin inhibited inflammatory response in fructose-induced THP cells, macrophage-like cells, via suppressing p300 histone acetyltransferase. The modulation by curcumin was characterized by decreased HAT activity and reduced p300 and acetylated CBP/p309 gene expression [149]. Moreover, curcumin inhibited the triggering receptors expressed on myeloid cells 1 (TREM-1), an amplifier of the immune response, via epigenetic modulation. The binding of NF-κB p65 to TREM-1 was attenuated by curcumin through inhibition of p300 activity in the TREM-1 promotor region [150]. Curcumin induced the receptor activator of nuclear factor-kappa B (RANK) expression through epigenetic reactivation of RANK in human glioblastoma cells [151], where activation of RANK by ligand (RANKL) played a crucial role in suppressing inflammatory response in microglial cells [152]. A study demonstrated that curcumin decreased the activity of p300/CBP histone acetyltransferase in CCI rats, which in turn decreases the spinal expression of BDNF and COX-2 [21]. The reduction in these mediators was consistent with the ability of curcumin to reduce pain hypersensitivity.

On the other hand, there has been growing interest in developing analgesic drugs that modulate inflammasomes. In chronic pain models, inflammasome is activated as indicated by the upregulation of numerous inflammasome markers, such as NALP1, caspase-1, and adaptor protein ASC [22,153]. Moreover, in mice with spinal cord injury, both microglia and astrocytes were activated with an increased release of IL-1β and caspase-1 [154]. Furthermore, the association of NLRP3 inflammasomes with neuroinflammation has been declared in fibromyalgia and CRPS [155]. Several studies reported that NLRP3 inhibitors ameliorate pain in numerous animal pain models [156,157]. MCC950, an NLRP3 inhibitor, attenuated pain behaviors in cancer-induced bone pain and multiple sclerosis-associated chronic pain. In addition, in mice with spinal cord injury, MCC950 decreased spinal neuroinflammation, as indicated by decreased TNF-α, IL-1β, and interleukin-18 (IL-18) via the blockage of the NLRP3 inflammasome [157]. Mechanistically, MCC950 blocks the production of the end products of this pathway, IL-1β and IL-18. IL-1β and IL-18 are mediators that play an essential role in pain transmission in the spinal cord; their production is due to the activation of the NLRP3 inflammasome.

A study of peripheral immune cells demonstrated that curcumin modulated NLRP3 inflammasome via the inhibition of contributing factors, such as the production of ROS and cathepsin B, K^+^ efflux, and purinergic activation [158,159]. On the other hand, curcumin can also inhibit NF-κβ activity, which is the first stage of the inflammasome processes [159]. In microglial cells, curcumin inhibited Ca^2+^ accumulation along with inflammatory response via suppressing P2 × 7 receptor activation [160], in which this receptor is an upstream pathway of NLRP3 inflammasome [161]. In a rat model of epilepsy, curcumin attenuated the expression of IL-1β, NLRP3, cleaved caspase-1 assessed in rats’ brains [162]. In a study on stressed rats, curcumin blocked the P2X7-NLRP 3 pathway and the transformation of pro-IL-1β to mature IL-1β [163]. In addition, in a mouse model of neuropathic pain, curcumin inhibited NALP1 inflammasome via JAK2-STAT3 signaling in astrocytes, suppressing the expression of IL-1β [22].

## 9. Physical and Pharmacokinetic Properties of Curcumin

### 9.1. Solubility and Stability

The therapeutic potential of curcumin is hindered by its low aqueous solubility (3.12 mg/L at 25 °C) and chemical instability [28,34]. Curcumin is stable in acidic conditions but not in neutral or basic conditions wherein it degrades to vanillin, ferulic acid, and vanillic acid under physiological conditions [29,30]. Moreover, it is a relatively unstable compound, which degrades in the presence of light [31]. These properties limit the application of curcumin as a bioactive agent.

### 9.2. Absorption

After oral administration, curcumin can be absorbed through intestinal segments, including the duodenum, jejunum, and ileum (Table 4). A study in animals found higher curcumin absorption in the duodenum than the jejunum and ileum segments of the intestine [164,165]. In the Caco-2 permeability assay, the permeation rate of curcumin was 5.14 × 10^−8^ cm/s [166]. In addition, other studies found the permeation rate of curcumin at 7.1–8.4 × 10^−6^ cm/s [167,168]. Moreover, Caco-2 cell permeability rates at <1–2, ~2–10, > 10 × 10^−6^ cm/s correspond to low, moderate, and high transcellular absorption in vivo, respectively [169]. Here, the permeability rate of curcumin ranged from 5.14 × 10^−8^ to 8.4 × 10^−6^, indicating a low to moderate transcellular absorption of curcumin in vivo.

### 9.3. Metabolism and Elimination

The rapid metabolism of curcumin is identified as one of the major factors that limit the use of curcumin as a therapeutic agent (Table 4). After curcumin is absorbed, successive reduction in the four double bonds present in the heptadiene-3-5, dione system of curcumin produces several active metabolites. Metabolism of curcumin occurs via several pathways, including the reduction by alcohol dehydrogenase to tetra- and hexa-hydrocurcumin in the liver and microsomal enzyme to di- and octa-hydrocurcumin. These reduced metabolites and curcumin then encounter glucuronidation and produces curcumin glucuronide and curcumin sulfate [170]. Metabolites of curcumin include curcumin-glucuronoside, curcumin sulfate, dihydrocurcumin-glucuronoside, tetrahydrocurcumin-glucuronoside, tetrahydrocurcumin, hexahydrocurcumin and hexahydrocurcuminol, and hexahydrocurcumin glucuronide [171,172,173,174]. Consequently, intestinal and hepatic metabolism of curcumin limits its oral bioavailability, which restricts the therapeutic potential of oral curcumin [175]. Moreover, curcumin can be detected in the urine and bile and is highly excreted through feces (75–90%) [173,174,176,177].

**Table 4:** Summary of absorption, distribution, metabolism, and elimination of curcumin.

Model	Dose/Concentration	Main Finding	Ref
Absorption			
Caco-2 permeability assay	20 μg/mL	Permeation rate 7.1 × 10^−6^ cm/s	(Yu and Huang, 2011) [168]
Caco-2 permeability assay	20 μg/mL	Permeation rate 8.4 × 10^−6^ cm/s	(Yu and Huang, 2012) [167]
Reverted rat gut sacs	100 μg/mL	Amount of curcumin in the serosal fluid of the jejunum > duodenum and ileum	(Y.-M. Tsai et al., 2012) [178]
		comparable amount of curcumin in sac tissue of duodenum, jejunum, and ileum	
Caco-2 permeability assay	30 μg/mL	Permeability coefficient 5.14 × 10^−8^ cm/s	(J. Wang et al., 2015) [166]
In vivo biodistribtion in rats	70 mg/kg	Curcumin was absorbed through intestinal segments, including the duodenum, jejunum, and ileum	
In situ single-pass intestinal perfusion test (SPIP)	5 µg/mL curcumin	Permeability coefficient of curcumin in duodenum > jejunum and ileum	(H. Ji et al., 2016) [164]
Rat intestinal perfusion study	40 μg/mL curcumin	Absorption rate and effective permeability of curcumin in the duodenum > jejunum and ileum	(Tian, Asghar, Wu, Chen et al., 2017) [165]
Distribution			
Male albino rats	400 mg curcumin	Portal blood, stomach, intestine, liver, and kidney	(Ravindranath and Chandrasekhara, 1980) [179]
Female BALB/c mice	100 mg/kg body weight, i.p	Plasma, liver, kidneys, spleen, brain, and intestines 1 h after i.p. administration	(Pan, Huang, and Lin 1999) [171]
Male Wistar albino rats	340 mg/kg	Intestinal mucosa, liver, kidney, and heart	(Marczylo, Steward, and Gescher 2009) [174]
Male ICR mice	20 and 400 mg/kg. p.o.	Plasma and central nervous system (brain and spinal cord)	(Szymusiak et al., 2016) [87]
Metabolism (metabolites)			
In vitro: hepatocytes or liver microsomes	0.1–5 μg/mL	60%–90% of curcumin was metabolized within 30 min	(Wahlström and Blennow 1978) [176]
In vitro hepatocytes	100 μM	Hexahydrocurcumin and hexahydrocurcuminol	(Ireson et al., 2001) [172]
Male Sprague–Dawley rats	0.6–12 mg, i.v	glucuronides of tetrahydrocurcumin and Hexahydrocurcumin, dihydroferulic acid, and ferulic acid	(Holder, Plummer, and Ryan 1978) [173]
Female BALB/c mice	100 mg/kg body weight, i.p	Curcumin-glucuronoside, dihydrocurcumin-glucuronoside, tetrahydrocurcumin-glucuronoside, and tetrahydrocurcumin	(Pan, Huang, and Lin 1999) [171]
Female F344 rats	500 mg/kg, p.o and 40 mg/kg, i.v.	Major: curcumin glucuronide and curcumin sulfate minor: hexahydrocurcumin, hexahydrocurcuminol, hexahydrocurcumin glucuronide	(Ireson et al., 2001) [172]
Male Wistar albino rats	340 mg/kg, p.o.	Phenolic glucuronides and alcoholic glucuronides (plasma and urine)	(Marczylo, Steward, and Gescher 2009) [174]
Human	Curcuminoids, 3.6 g/d for 29 days, p.o	Curcumin glucuronide and curcumin sulfate (plasma and urine)	(R.A. Sharma et al., 2004) [177]
Elimination			
Sprague–Dawley rats	1 g/kg	75% of curcumin excreted in the feces, the undetectable amount in urine	(Wahlström and Blennow 1978) [176]
Sprague–Dawley rats	0.6 mg/dose	-[^3^H]-curcumin metabolites—89.4% (feces) 6.3% (urine)	(Holder, Plummer, and Ryan 1978) [173]
		High extent of biliary excretion of curcumin	
Male Wistar albino rats	340 mg/kg, p.o.	2.0 ng/mL of curcumin (urine)	(Marczylo, Steward, and Gescher 2009) [174]
Human	Curcumuminoids, 3.6 g/d for 29 days, p.o	Curcumin and its metabolites (urine and feces)	(R.A. Sharma et al., 2004) [177]

### 9.4. Bioavailability and BBB Penetration

The low oral bioavailability of curcumin is another important clinical concern. Relatively low plasma concentrations of curcumin have been observed in the patient administered with considerably high doses of curcumin. This is associated with poor absorption, and rapid metabolism, and excretion of curcumin from the body.

The details of the pharmacokinetic properties of curcumin are summarized in Table 4 and Table 5. A study in mice showed that oral administration of curcumin at a dose of 1000 mg/kg resulted in a maximum plasma concentration of 220 ng/mL. In the same study, intraperitoneal administration of 100 mg/kg curcumin exhibited a higher maximum plasma concentration of curcumin (2250 ng/mL) compared to oral administration at the dose of 1000 mg/kg [171]. As shown in Table 4, several studies in rodents showed the concentration of curcumin in the plasma at the range of 0.6–2120 ng/mL after oral administration at 20–1000 mg/kg. The intravenous administration of curcumin (10 mg/kg) resulted in a plasma concentration of 360–8820 ng/mL. The oral bioavailability of curcumin was reported to be at the range of 0.21–4.74% [180,181,182,183,184].

Furthermore, several studies in humans have also been performed. In cancer patients, 1.77 μM of curcumin was found in the plasma 1–2 h after administering curcumin at 8 g/day for 3 months [185]. In another study, curcumin consumption by colorectal cancer patients at 3.6 g/day yielded a plasma concentration of 4.1 ng/mL after 1 h of administration [177]. Conversely, the administration of *Curcuma* extract containing 8.18% curcumin to colorectal cancer patients for 1 month showed no detectable curcumin in the plasma [186] (Table 5). However, several curcumin formulations have been developed with promising improvements of the bioavailability compared to native curcumin, such as Meriva^®^ Theracurmin™ NovaSol^®^ [187,188,189]. However, previous pharmacokinetic studies demonstrate pronouncedly variable data between studies, which could be due to differences in dose, preparation, and purity of curcumin and detection devices.

Since the pathological state of progressive chronic pain occurs in the CNS, drugs targeting the brain and spinal cord should enter the bloodstream, have a long half-life, and possess the ability to penetrate the BBB to attenuate neuroinflammation. However, only 1.4–41.1 ng/g of curcumin was found in the brain tissues of naïve rodents after administrating 20–400 mg/kg dose of curcumin orally (Table 6). In contrast, there was no detectable curcumin in the brain tissues 30–120 min after oral administration of 50 mg/kg of curcumin [190]. The intraperitoneal route showed a high degree of curcumin concentration in the brain compared to the oral route. The intraperitoneal administration of 100 mg/kg of curcumin in rodents resulted in 410–5010 ng/g curcumin in the brain tissues. For intravenous administration, 2–5 and 25.7 ng/g of curcumin content in the brain were found after 5 mg/kg and 10 mg/kg of curcumin administration, respectively. In another study, curcumin was observed in several brain regions, such as the hippocampus, cerebral cortex, cerebellum, striatum, and brain stem, after intraperitoneal administration [191]. Interestingly, one study found that curcumin can reach the spinal cord, a target organ of exposure for spinal neuroinflammation, in which 20 mg/kg and 400 mg/kg curcumin administered orally, resulting in curcumin concentrations of 23.49 ng/g and 129.16 ng/g, respectively [87]. In one study, 108.3 ng/g of curcumin in the brain provided pharmacological effect in lipopolysaccharide (LPS)-induced neuroinflammation in mice [192]. In the same study, much higher curcumin content was found in the LPS-treated mice compared to the normal mice, where normal and LPS-treated mice produced curcumin content in the brain at 41.1 ng/g and 108.3 ng/g, respectively [192]. This result is realistic, where several studies reported that inflammation causes disruption in the blood-brain barrier [193]. Pain conditions have been reported to increase the permeability of the blood-brain barrier and blood-spinal cord barrier in rodent models of inflammatory and neuropathic pain [194,195,196]. As such, the curcumin content in the brain of animals with pain conditions could be much higher than that of normal mice. Furthermore, the use of other routes to administer curcumin is a feasible alternative to oral administration to bypass extensive first-past metabolism. A new application of curcumin by intrathecal administration has been performed in CFA rats in which intrathecally administered curcumin targets the region of neuroinflammation more specifically to control pain. The results demonstrated a substantial decrease in pain hypersensitivity and inflammation in the spinal cord [17]. In addition, several other options such as drug combination, formulation, and prodrug might be plausible to improve the pharmacokinetic properties of curcumin, which are detailed in the chapter below.

**Table 5:** Plasma pharmacokinetics of curcumin.

No	Species	Route	Dose (mg/kg)	Pharmacokinetic Parameters				Ref
				Plasma Concentration (ng/mL)	t_max_ (min)	t _1/2_ (min)	F (%)	
	Mice							
1	Male ICR mice	p.o	20	0.60 ± 0.44	15	N/A	N/A	(Szymusiak et al., 2016) [87]
			400	79.82 ± 49.00	3			
2	Male C57BL/6 mice	p.o	25	14 ± 3	120	N/A	N/A	(C. Wang et al., 2015) [197]
3	BALB/C mice	p.o	150	800 ± 200	60 ± 34.2	N/A	N/A	(S. Kumar et al., 2016) [198]
4	Female BALB/c mice	p.o	1000	220		N/A	N/A	(Pan, Huang, and Lin 1999) [171]
		i.p.	100	2250				
	Rats							
1	Male Sprague–Dawley rats	p.o	20	82 ± 8	30	N/A	N/A	(C. Liu et al., 2018) [199]
2	Male Sprague–Dawley rats	p.o	50	290 ± 110	60	N/A	N/A	(Baek and Cho 2017) [200]
3	Male Sprague–Dawley rats	p.o	50	22 ± 6	N/A	N/A	2.60 ± 1.03	(Tian, Asghar, Wu, Kambere Amerigos et al., 2017) [180]
4	Male Sprague–Dawley rats	p.o	50	5.08 ± 1.18	60	541.11 ± 395.78	N/A	(Yutong Wang et al., 2017) [201]
5	Sprague–Dawley rats	p.o	50	109.84 ± 85.89	69 ± 44.4	N/A	N/A	(J. Wang, Ma, and Tu 2015) [166]
6	Male Wistar rats	p.o	50	2120 ± 340	34.8 ± 12	570 ± 12	N/A	(H. Ji et al., 2016) [164]
7	Male Wistar rats	p.o	50	87.06 ± 24.02	39.6 ± 3.6	29.4 ± 4.8	N/A	(Wan et al., 2012) [202]
8	Albino Wistar rats	p.o	50	9.58 ± 0.4	30 ± 0.0	75 ± 2.25	N/A	(Chaurasia et al., 2015) [203]
9	Male Wistar rats	p.o	50	4.07 ± 0.56	30	66.54 ± 7.44	N/A	(Khalil et al., 2013) [204]
10	Male Sprague–Dawley rats	p.o	100	470 ± 180	60	N/A	N/A	(Peng et al., 2018a) [205]
11	Male Sprague–Dawley rats	p.o	100	1550 ± 210	102 ± 16	74.2 ± 5.9	4.73	(X. Xie et al., 2011) [181]
12	Male Sprague–Dawley rats	p.o	100	21.6 ± 3.6	60	246 ± 18	N/A	(Shukla et al., 2017) [206]
13	Male Sprague–Dawley rats	p.o	100	35 ± 8.0	80 ± 10	207 ± 94	0.9	(Onoue et al., 2010) [182]
14	Wistar rats	p.o	100	126 ± 13.56	60	70.2 ± 2.4	N/A	(A. Kumar et al., 2016) [207]
15	Male Sprague–Dawley rats	p.o	150	1480 ± 30	15 ± 0.00	304.2 ± 24.6	N/A	(Q. Zhang et al., 2018) [208]
16	Male Sprague–Dawley rats	p.o	250	90.3 ± 15.5	30	N/A	N/A	(Shaikh et al., 2009) [209]
17	Male Sprague–Dawley rats	p.o	250	32.29 ± 14.93	34.8 ± 12	28.2 ± 8.4	N/A	(Joshi et al., 2013) [210]
18	Male Sprague–Dawley rats	p.o	500	60 ± 10	41.7 ± 5.4	44.5 ± 7.5	1%	(Yang et al., 2007) [183]
19	Male Wistar rats	p.o	500	3.2 ± 1.4	30	N/A	N/A	(Teixeira et al., 2016) [211]
20	Sprague–Dawley rats	p.o	1000	950 ± 120	84 ± 33	184.8 ± 75.6	N/A	(Hu et al., 2015) [212]
21	Male Sprague–Dawley rats	p.o	1000	28 ± 10	45	N/A	N/A	(Y.-M. Tsai et al., 2012) [178]
22	Male Sprague–Dawley rats	p.o	1000	15 ± 12	50 ± 32	95 ± 35	N/A	(Chang et al., 2013) [213]
23	Male Sprague–Dawley rats	p.o	1000	22 ± 2	N/A	N/A	0.21	(Y.-M. Tsai et al., 2011) [184]
24	Wistar Albino Rats	p.o	1000	258.64	103.2	76.8	N/A	(Gupta and Dixit 2011) [214]
25	Sprague–Dawley rats	p.o	1000	830 ± 830	180.0 ± 60.0	61.24 ± 15.17	N/A	(Hu et al., 2012) [215]
26	Male Sprague–Dawley rats	i.v.	10	360 ± 50	N/A	28.1 ± 5.6	N/A	(Yang et al., 2007) [183]
27	Male Sprague–Dawley rats	i.v.	10	8820 ± 110	3	N/A	N/A	(X. Xie et al., 2011) [181]
28	Male Sprague–Dawley rats	i.v.	10	4200 ± 1800	N/A	N/A	N/A	(Y.-M. Tsai et al., 2011) [184]
	**Species**	**Route**	**Form of Curcumin**			**Dose** **(Cur/Cur equivalent)**	**Plasma Conc.** **(ng/mL)**	
1	Human	p.o	Curcumin			4000–8000 mg/day (3 months)	187.9–652	(A.L. Cheng et al., 2001) [185]
2	Human	p.o	Curcuminoid capsules (8.2% curcumin)			36 and 180 mg curcumin/day (29 days)	Undetectable	(R A Sharma et al., 2001) [186]
3	Human	p.o	Curcuminoid capsules (90% of curcumin)			3.6 g/day	4.1 ± 0.2	(Sharma, R.A et al., 2004) [177]
4	Healthy human	p.o	Standardized curcuminoid mixture			1295 mg	9.0 ± 2.8	(Cuomo et al., 2011) [187]
		p.o	Lecithin formulation of standardized curcuminoid mixture (Meriva)			297 mg	50.3 ± 12.7	
5	Healthy human	p.o	Curcumin			30 mg	1.8 ± 2.8	(Sasaki et al., 2011) [188]
		p.o	Curcumin colloidal nanoparticles (Theracurmin™)			30 mg	29.5 ± 12.9	
6	Healthy human	p.o	Curcumin			410 mg	2.6 ± 4.9	(Schiborr et al., 2014) [189]
		p.o	Liquid micelles of curcumin (NovaSol^®^)			410 mg	1189.1 ± 518.7	
		p.o	micronized curcumin			410 mg	15.3 ± 8.9	

Abbreviations: p.o, oral administration; i.v, intravenous administration; i.p, intraperitoneal administration; N/A, not available t_max_, time of maximum concentration observed; t _½_, terminal half-life; F (%), oral bioavailability.

**Table 6:** Brain pharmacokinetic of curcumin.

No.	Species	Route	Dose	Concentration			Ref
				Brain (ng/g)	Spinal Cord (ng/g)	Plasma (ng/mL)	
1	NMRI mice	p.o	50 mg/kg	Undetectable	N/A	N/A	(Schiborr et al., 2010) [190]
	C57BL/6 mice	i.p	100 mg/kg	4160–5010			
2	Wistar rats	p.o	200 mg/kg	1.40 ± 0.80	N/A	N/A	(IM, K.; Maliakel et al., 2015) [216]
3	Male ICR mice	p.o	400 mg/kg	30.32 ± 3.10	129.16 ± 63.12	79.82 ± 49.00	(Szymusiak et al., 2016) [87]
			20 mg/kg	2.03 ± 0.69	23.49 ± 11.57	0.60 ± 0.44	
4	Male C57BL/6 mice	p.o	50 mg/kg/d for 2 days	41.1 ± 6.7	N/A	8.2 ± 1.8	(Sorrenti et al., 2018) [192]
	Male C57BL/6 mice-induced by LPS	p.o.	50 mg/kg/d for 2 days	108.3 ± 25.8	N/A	4.8 ± 0.9	
5	Female BALB/c mice	i.p.	100 mg/kg	410 ± 10	N/A	N/A	(Pan, Huang, and Lin 1999) [171]
6	Kunming male mice	i.v.	10 mg/kg	25.7	N/A	N/A	(Sun et al., 2010) [217]
7	C57BL/6 mice	i.v.	5 mg/dose	2–5	N/A	2–5	(Dende et al., 2017) [218]

Abbreviations: BALB/c, Bagg Albino; p.o, oral administration; ICR, institute of cancer research; i.v, intravenous administration; i.p, intraperitoneal administration; LPS, lipopolysaccharides; NMRI, Naval Medical Research Institute; N/A, not available.

### 9.5. Drug-Interactions

Several isoforms of drug-metabolizing enzymes, including cytochrome P450, glutathione-S-transferase, and UDP-glucuronosyltransferase, have been demonstrated to be inhibited by curcumin and its derivatives. Inhibition of these enzymes could lead to an unintended increase in systemic bioavailability of drugs that are metabolized via these enzymes, including digoxin, acetaminophen, and morphine [219].

## 10. Strategies to Improve the Efficacy of Curcumin on Neuroinflammation-Driven Chronic Pain

Over recent years, extensive efforts have been made to overcome the aforementioned limitations of curcumin, such as nanotechnology, prodrug approach, and drug combination approach.

### 10.1. Curcumin Nanoparticles

Ideally, the oral delivery of nano-formulation of curcumin can enhance its solubilization in the gastrointestinal milieu and improve delivery through the enterocytes. In addition, nano-formulated curcumin, which targets the blood-brain barrier, could be promising due to its potential direct activity on glial cells. It was shown that curcumin nanoparticles decreased neuroinflammation-mediated chronic pain and demonstrated better pharmacological responses than curcumin [102,220]. Despite the efficacy of curcumin in treating chronic pain, its physicochemical and pharmacokinetic properties remain challenging. Therefore, the development of curcumin nanoparticles with better pharmacokinetic properties and efficacy has gained much attention.

As inflammatory suppressors, curcumin-loaded solid lipid nanoparticles were also more efficacious than curcumin. For example, curcumin-loaded solid lipid nanoparticles were found to attenuate NO and the mRNA level of iNOS, COX-2, TNF-α, IL-1β, and IL-6 in LPS-stimulated BV-2 cells [221]. Moreover, in rats with spinal cord injury, nano-formulated curcumin with lipid-based discoidal nanoparticles effectively reduced spontaneous pain concomitantly with a decreased glial scars and inflammatory responses [220]. In a mouse model of neuropathic pain, curcumin-loaded poly (d,l-lactide-co-glycolide) nano-vesicles (i.t) decreased mechanical and thermal hypersensitivities more effectively than curcumin because of its ability to inhibit the enriched TNF-α, IL1-β, IL-6, and BDNF in the spinal cord of mice [102]. In addition, a formulation of PLGA-curcumin nanoparticles inhibited thermal and mechanical opioid-induced hyperalgesia to a greater extent compared to curcumin. This inhibition was mediated via the inhibition of CaMKIIα activation [103], in which CaMKIIα is one of the regulators in the development of inflammatory pain [222]. In addition, curcumin-loaded nanoparticles had an enhanced ability to penetrate the BBB. Tail vein injection of curcumin-loaded nanoparticles prepared by a single emulsion method demonstrated the ability to cross the BBB and suppressed microglial activation and the release of MMP9, TNF-α, and IL-1β in the mouse model of cerebral ischemia/reperfusion [223].

### 10.2. Curcumin Prodrugs

Aqueous solubility, one of the essential properties of an orally administered drug, is deficient for curcumin. Low solubility and rapid metabolism of curcumin result in low and variable oral bioavailability, leading to low oral efficacy [33,34]. The prodrug approach is used to improve the physicochemical and pharmacokinetic properties of curcumin [224]. Curcumin prodrug is a pharmacologically inactive form of curcumin that becomes activated via chemical or enzymatic processes in the body after ingestion.

Several curcumin prodrugs, such as curcumin–amino acid prodrugs, curcumin–NSAID prodrugs, and curcumin conjugated with either diethyl disuccinate or glutaric acid, have been developed to overcome the limitations of curcumin. Curcumin–amino acid prodrugs are a series of prodrugs that have been formulated in combination with several amino acids. Some of the curcumin–amino acid prodrugs exhibited better analgesic efficacy in visceral and thermal nociceptive pain models and anti-inflammatory activity in rodents induced by carrageenan than that of the parent curcumin [225]. In an in vitro study, curcumin prodrugs containing alanine, cysteine, serine, and valine promoieties showed better antioxidant activity than curcumin [226].

A codrug, or mutual prodrug, combines two active drugs. Codrugs were also developed to improve the efficacy of oral curcumin. In this approach, curcumin conjugated with NSAIDs (curcumin–NSAID prodrugs) was more effective than the analgesic efficacy of curcumin. For example, the flufenamic acid–curcumin codrug demonstrated better anti-inflammatory activity than the parent drug [227]. In another study, the transdermal application of curcumin combined with either salicylic acid or salsalate exhibited improved anti-inflammatory activity via the suppression of IL-1β, IL-6, and TNF-α over curcumin and NSAIDs alone [228].

We have shown that the curcumin diethyl disuccinate prodrug has improved chemical stability and cell permeation and exhibits greater thermal and visceral nociceptive activity [229,230]. Although several studies have reported the efficacy of curcumin in pain treatment, little is reported about its efficacy in neuroinflammation-associated pain. We have formulated curcumin diglutaric acid (CurDG), a curcumin prodrug that is more stable and soluble than curcumin. CurDG was found to reduce peripheral inflammation and spinal neuroinflammation to a greater extent than curcumin in a mouse model of neuropathic pain. This result was in line with the ability of CurDG to reduce behavioral pain hypersensitivity, both mechanical and thermal hypersensitivities [101]. The same effect was observed with the treatment of curcumin diethyl diglutarate (CurDDG), where it improved cytokine level and pain-like behaviors to a greater extent than curcumin in a mouse model of neuropathic pain [83].

### 10.3. Drug Combination Approach

Another commonly used approach to overcome the limitations of curcumin is the use of drug combinations. Here curcumin is administered with other synthetic or natural compounds. The administration of curcumin with an adjuvant is expected to elicit a more favorable therapeutic outcome due to the synergistic interaction between the compounds.

For example, curcumin administered with diclofenac sodium showed synergistic interaction in the formalin test, indicating anti-inflammatory synergism [231]. Our recent publication demonstrated the synergistic interaction between curcumin and pregabalin in acute nociceptive pain [232]. However, the mechanism underlying the synergism between curcumin and diclofenac or pregabalin needs to be further evaluated. In addition, limited data are available for the synergistic interaction of curcumin with any adjuvants in chronic pain models. Therefore, future studies are recommended to assess the possibility of improving the efficacy of curcumin in treating neuroinflammation-driven chronic pain with adjuvants.

## 11. Potential Risks and Adverse Effects of Curcumin

Curcumin has well-established records of being safe, tolerable, and non-toxic. For example, rodents treated with turmeric oleoresin (79–85%) of curcumin for a short (13 weeks) or long period (2 years) showed no sign of significant toxicity and carcinogenesis at doses lower than 1300 mg/kg [233]. Curcumin was also proven to be safe and well-tolerated at a dose of 12 g/day in a clinical study [25]. Moreover, curcumin has been classified as a “generally recognized as a safe” compound by the U.S. Food and Drug Administration (FDA) [26]. Regardless of these authentic safety records of curcumin, a few negative adverse effects have been reported specially when large doses were used to gain “adequate systemic exposure”. In one study, seven out of 24 participants treated with curcumin at the dose of 500–1200 mg experienced diarrhea, yellow stool, headache, and rash within 72 h post-treatment [25]. Moreover, another phase I clinical trial reported the development of nausea, diarrhea, and increased levels of serum alkaline phosphate and lactate dehydrogenase with the treatment of curcumin at 0.45–3.6 g/day for one to four months [177]. Further, GI toxicity identified as abdominal fullness and pain has also been reported in the advanced pancreatic cancer patients treated with oral 8000 mg curcumin divided into two daily doses for three to four weeks. In addition, two out of 15 patients with primary sclerosing cholangitis treated with 750 mg twice a daily dose of curcumin developed nausea and headache [234]. Apart from that, a recent case study reported the development of turmeric supplement-induced autoimmune hepatitis, which has to be further investigated to confirm whether the hepatotoxicity is associated with curcumin itself or the other ingredients or contaminants present in the supplement [235]. Nevertheless, most of the studies reporting the safety and toxicity of curcumin have been conducted for a short period. Hence, further investigations on the utility of curcumin as a long-term analgesic agent are warranted to ensure the safety of curcumin with chronic administration.

## 12. Conclusions and Future Perspectives

Multiple lines of evidence show that spinal neuroinflammation mediates chronic pain progression and may be a target for pharmacological interventions. Targeting glial cell-mediated spinal neuroinflammation has gained increasing scientific interest during the past few decades [236]. Several in vitro and in vivo studies have demonstrated that curcumin can reduce the severity of neuroinflammation in chronic pain models. The anti-inflammatory activity of curcumin in microglia and astrocytes is attributed to its ability to inhibit various pro-inflammatory mediators by impeding inflammatory cascades such as MAPK, NF-κβ, and JAK-STAT pathways, consequently reducing pain hypersensitivity and spontaneous pain. Curcumin also increases endogenous anti-inflammatory mediators; thus, it may function as an immunomodulator. However, despite the significant analgesic activity of curcumin, it remains unresolved whether the main effect of curcumin is by its direct effects on the spinal cord and brain or indirect effect on other sites of action. Curcumin was found to be at a very low level in the brain, even undetectable in some studies. Therefore, it is crucial to determine whether the main activity of curcumin is direct or indirect. In our perspective, curcumin is believed to act not only in the CNS but also in the other sites of action. Curcumin has a direct action on sensory neurons (nociceptors) via desensitizing TRPV1 and modulating purinergic and CX3CR1receptors in the dorsal root ganglia [88,89,90,91]. Curcumin also inhibited peripheral immune cells [95] and modulated gut microbiota [98,99]. Therefore, the decreased immune response in the spinal cord can also be due to the indirect actions of curcumin on the peripheral nerves, peripheral immune cells, and gut microbiota instead of its direct activity on the spinal cord; modulating peripheral nerves, peripheral immune cells, and gut microbiota potentially limits the activation of the spinal glia by inhibiting neuron-glia, peripheral-central neuroimmune, and gut-neuroimmune interactions.

On the other hand, poor physiochemical properties, low bioavailability, low curcumin content in the brain, and a lack of clinical trial data are significant factors limiting the feasibility of curcumin as a therapy. As such, the use of higher oral doses and other routes of administration are required to compensate for the poor physicochemical and pharmacokinetic properties of orally administered curcumin. In addition, developing curcumin prodrugs and encapsulated nanoparticles and combining curcumin with other compounds are strategies under examination to improve the stability, potency, and bioavailability of curcumin. However, increased curcumin bioavailability and its content in the CNS by nanoparticles, prodrugs, and adjuvants might cause adverse effects. Low side effects achieved by curcumin might be due to its low bioavailability, while increasing its bioavailability might cause a high risk of off-target effects and narrows its therapeutic window. Curcumin is a molecule that interacts with a large number of cellular, protein, and receptor targets, making it a natural product with polypharmacological characteristics. Hence, it is essential to explore off-target potencies of curcumin formulations, either prodrugs, drug combinations, or nanoformulations, and their potential side effects to ensure the safety of those formulations.

The content of active metabolites of curcumin in the brain and spinal cord and its potential efficacy after administration of curcumin should also be characterized since oral, intraperitoneal, and intramuscular administration of tetrahydrocurcumin, a curcumin metabolite, was able to penetrate to the brain and effectively inhibited the inflammatory response in the central nervous system [237]. Hence, it will be beneficial to denote and clarify whether the efficacy arises only from the parent molecule or its metabolites after being administered native curcumin in animal models of chronic pain. One approach in drug development known as metabolism-activated multi-targeting (MAMUT), where parent drug synergistically interacts with its metabolites, has been introduced [238]. Hence, it will be worthwhile to investigate whether curcumin possesses the MAMUT in terms of alleviating neuroinflammation-induced chronic pain. Lastly, future clinical studies are necessary to further investigate the activity of curcumin on neuroinflammation-driven chronic pain.

## Data Availability

Data sharing not applicable.
